# Expression of neuropilin-1 on in vivo induced regulatory T cells

**DOI:** 10.1186/2051-1426-1-S1-P167

**Published:** 2013-11-07

**Authors:** Christopher J Nirschl, Christina Ceccato, Angela Alme, Brian Francica, Charles G Drake

**Affiliations:** 1 Oncology, Johns Hopkins School of Medicine, Baltimore, MD, USA; 2 Immunology, Johns Hopkins School of Medicine, Baltimore, MD, USA; 3 Urology, Johns Hopkins School of Medicine, Baltimore, MD, USA

## 

One of the current questions surrounding CD4 T regulatory cells (Tregs) is the role of natural and induced Tregs in tumor tolerance. Natural Tregs are CD4 T cells that leave the thymus expressing FoxP3 and displaying regulatory potential. Induced Tregs leave the thymus as a naïve CD4 T cell (FoxP3-), but are skewed by conditions encountered during antigen recognition to express FoxP3 and gain regulatory function. Several groups have recently suggested that Neuropilin-1 may be a suitable marker for natural Tregs. To examine the ability of neuropilin-1 to identify natural versus in vivo induced Tregs, we bred a CD4 T cell receptor transgenic mouse specific for HA (6.5) onto a Rag-2 -/- background. The resultant 6.5 Rag -/- mice have no Tregs (as defined by FoxP3 expression), as has been noted for other TCR transgenic models, and express no neuropilin-1 (or CD25) prior to manipulation. To test whether neuropilin-1 was expressed on in vivo induced Tregs we performed a series of adoptive transfer studies into A) non-transgenic hosts, followed by low dose i.v. HA peptide administration B) self antigen expressing animals (C3-HAlow) or C) tumor antigen expressing mice (established HA expressing 4T1 mammary carcinomas). Our data show that in vivo induced Tregs clearly express neuropilin-1 under these various conditions. Furthermore, neuropilin-1 expression was found on both FoxP3+ and FoxP3- populations in all three models of in vivo induced Tregs, suggesting that neuropilin-1 is expressed on activated cells. Consistent with this notion, we found that neuropilin-1 was strongly upregulated within the first several divisions on 6.5 CD4 T cells when adoptively transferred into a host receiving HA expressing Vaccinia Virus. CD25 expression was more closely associated with FoxP3 expression than neuropilin-1: In the low dose i.v. peptide model, neuropilin-1+ CD25+ 6.5 CD4 T cells were significantly enriched for FoxP3 and Helios expression, whereas neuropilin-1+ CD25- 6.5 CD4 T cells were not. Tumor induced 6.5 CD4 Tregs also expressed low levels of neuropilin-1, even though the majority of 6.5 CD4 T cells were not induced to express FoxP3 by tumor antigen recognition. These data strongly support the notion that neuropilin-1 is not a specific marker of natural Tregs in mice, and further suggest that the majority of FoxP3+ TILS are likely natural Tregs, rather than induced Tregs.

